# Benchmark for Quantitative Global and Redox Proteomics Analysis by Combining Protein-Aggregation Capture and Data Independent Acquisition

**DOI:** 10.1021/acs.analchem.5c03294

**Published:** 2025-09-03

**Authors:** Ana Martinez-Val, Samuel Lozano-Juárez, Jorge Lumbreras Burgueño, Irene Rodríguez, Marinela Couselo-Seijas, Ana Simón-Chica, Carlos Galán-Arriola, Rodrigo Fernández-Jiménez, Estefanía Núñez, Inmaculada Jorge, David Filgueiras-Rama, Borja Ibáñez, Jesús Vázquez

**Affiliations:** † Cardiovascular Proteomics Laboratory, 38799 Centro Nacional de Investigaciones Cardiovasculares Carlos III (CNIC), Madrid 28029, Spain; ‡ Centro de Investigación Biomédica en Red de Enfermedades Cardiovasculares (CIBERCV), Madrid 28029, Spain; § Centro Nacional de Investigaciones Cardiovasculares Carlos III (CNIC), Novel Arrhythmogenic Mechanisms Program, Madrid 28029, Spain; ∥ Translational Laboratory for Cardiovascular Imaging and Therapy, Centro Nacional de Investigaciones Cardiovasculares Carlos III (CNIC), Madrid 28029, Spain; ⊥ Cardiovascular Health and Imaging Laboratory, Centro Nacional de Investigaciones Cardiovasculares (CNIC), Madrid 28029, Spain; # Instituto de Investigación Sanitaria del Hospital Clínico San Carlos (IdISSC), Cardiology Department, Madrid 28040, Spain; ¶ Department of Cardiology, Hospital Universitario Clínico San Carlos, Madrid 28040, Spain; ∇ IIS-Fundación Jiménez Díaz Hospital, Madrid 28040, Spain; 9 Proteomics Unit, 38799 Centro Nacional de Investigaciones Cardiovasculares Carlos III (CNIC), Madrid 28029, Spain

## Abstract

Oxidative damage plays a critical role in various diseases including cardiovascular and neurological disorders. Thiol redox reactions, acting as oxidative stress sensors, influence protein structure and function. Redox proteomics, based on the differential alkylation of cysteine sites followed by mass spectrometry, enables the comprehensive analysis of thiol redox status in cells and tissues. However, these approaches require extensive sample manipulation and are not compatible with data-independent acquisition techniques. Here, we introduce PACREDOX, an innovative strategy based on protein aggregation capture (PAC), and demonstrate its compatibility with library-free DIA. Compared with traditional methods such as FASILOX, PACREDOX reduces preparation time and costs while maintaining thiol and proteome coverage. To enable library-free DIA, we corrected *in silico* spectral libraries in DIA-NN using experimental retention time data from methylthiolated-Cys peptides. PACREDOX with DIA was benchmarked against FASILOX in a myocardial infarction model, yielding the same biological insights, while enhancing peptide and protein coverage. Our results underscore the potential and efficiency of this methodology for studying oxidative damage. Overall, PACREDOX offers an automatable, high-throughput, and cost-effective strategy for redox proteomics.

## Graphical abstract

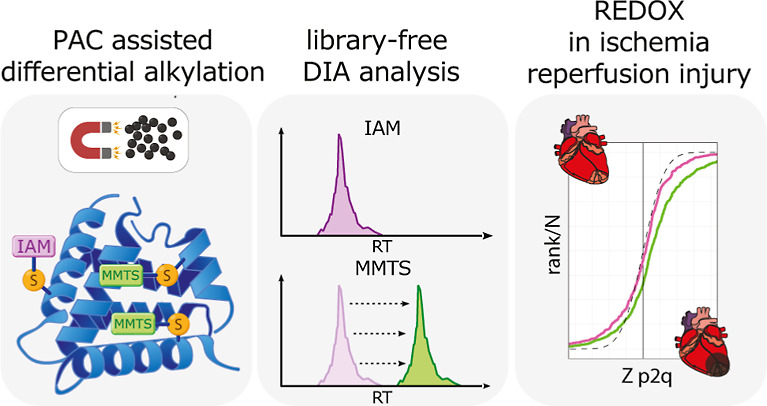

## Introduction

The study of redox post-translational modifications (PTMs) on Cys residues is essential to understand protein functionality and homeostasis. Redox PTMs, which alter proteins’ structural and functional properties, are produced when the thiol moiety in Cys is modified by a redox reaction. Such reactions can be induced by either reactive oxygen species (ROS), or specific enzymatic reactions. These modifications significantly impact cellular processes as they regulate protein stability, activity, and interactions. Given the complexity of redox PTMs, redox proteomics has emerged as a valuable approach to map these modifications on a systems level. Mass spectrometry (MS) has become the primary tool for large-scale analysis of redox-related protein modifications, allowing researchers to localize modifications at the amino acid level and to quantify relative abundance, thus providing comprehensive insights into redox biology.^1^

Redox reactions on free thiols (SH groups) on Cys residues may lead to a spectrum of oxidative modifications. Some of these modifications can be reverted by reductors and are so-called reversible. They include, among others, *S*-sulfenylation, *S*-nitrosylation, *S*-glutathionylation, disulfide bond formation, and *S*-polysulfidation. This reversibility enables the dynamic regulation of protein function and contributes to redox signaling. On the other hand, irreversible modifications, like *S*-sulfinylation and *S*-sulfonylation, often result in permanent structural changes, marking proteins for degradation or altering their interactions with other molecules.^2,3^

In conventional bottom-up proteomics, proteins are usually treated with reducing agents to optimize digestion with proteases, and thus reversible Cys modifications are lost. Several experimental strategies have been developed to circumvent this problem. A popular strategy is differential alkylation, which involves the selective reduction and tagging of different redox states. Initially, free thiol groups (corresponding to the basal or reduced Cys state) are usually blocked with an alkylating reagent to prevent further modifications. Subsequently, specific reductants are applied to the sample to reduce reversible oxidized forms, which are converted into free thiol groups; which are then subjected to a second alkylation step using a distinct reagent. This approach allows researchers to distinguish between different redox states and to tag specific modifications for further study.

For enhanced detection sensitivity, enrichment methods can be employed. OxICAT (isotope-coded affinity tags) employs thiol-reactive tags that facilitate both enrichment and quantification of Cys modifications.^4^ Resin-assisted capture (RAC), which uses thiolated sepharose beads, covalently captures proteins containing free thiols.^5^ Another strategy, CysPAT, uses a reagent containing a thiol-reactive group with a linker and a phosphate group, which allows for protein trapping through immobilized metal affinity chromatography (IMAC).^6^ Other techniques, such as GELSILOX and FASILOX, do not use enrichment steps but only require minor additions to conventional protocols and allow the analysis of different redox Cys states alongside with overall protein abundances, making them versatile tools to perform redox and conventional proteomics at the same time.^7,8^ In addition to these enrichment techniques, chemoselective probes have also been employed to detect specific redox PTMs with high specificity. These approaches involve the reaction of thiol redox forms with functionalized groups, such as biotin, which are then used to capture and enrich those modified peptides. Chemoselective probes have proven to be useful to study modifications such as *S*-nytrosylation^9^ or *S*-persulfidation.^10,11^ In contrast, direct detection approaches, which forego derivatization steps, have also emerged, allowing researchers to monitor redox PTMs with greater precision.^12,13^

In the recent years, the aggregation of proteins into particles have been proposed as a robust method for preparing samples for proteomics workflows, such as SP3 ^14^ or PAC.^15^ These protocols increase the throughput of sample preparation, but in contrast to in-solution digestion strategies, they also efficiently clean the sample. Additionally, compared to other sample preparation strategies, such as filter-based methods that also allow for solvent exchange, particle aggregation-based workflows are easier to streamline using magnetic racks and help avoid issues such as filter blockage in filter-assisted sample preparation (FASP).

From a mass spectrometry perspective, redox proteomics has traditionally relied on data-dependent acquisition (DDA) for quantification. However, recent advances have increased the adoption of data-independent acquisition (DIA) approaches. Techniques like oxSWATH utilize DIA for comprehensive quantification in redox proteomics; however, this technique initially relies on DDA strategies to build the spectral libraries required for DIA analysis.^16^ Library-free methods represent a promising area for future research; however, they remain largely unexplored in redox proteomics. Computationally predicted libraries, such as those generated by DIA-NN^17^ and Prosit,^18^ can predict fragmentation spectra of iodoacetamide (IAM)-modified peptides. However, they are limited to this specific alkylating agent, restricting their application to broader redox PTM analysis. Emerging tools such as directDIA in Spectronaut, which supports searches for various PTMs, offer potential solutions to this limitation. Nevertheless, access to such tools may be restricted due to licensing constraints.

In this work, we present an application of the protein aggregation capture (PAC)-based proteomics sample preparation method to study the thiol redox proteome. Furthermore, we demonstrate that DIA analysis can be performed without the need to construct experimental DDA libraries. Our approach enables redox proteomics using DIA, enhancing the depth of the thiol redox proteome while reducing the sample preparation time and subsequent costs.

## Experimental Section

### Experimental Design and Animal Models

#### Porcine Model of Long-Lasting Lone Persistent Atrial Fibrillation

Yucatan-Large White crossbred pigs were used to generate the long-lasting lone atrial fibrillation (AF) model as previously described.^19^ For this work, two independent samples were employed.

#### Porcine Model of Ischemia/Reperfusion

Castrated male Large White pigs weighing 30–40 kg were used for the pig ischemia/reperfusion (I/R) experiments as described.^12^ For this work, three independent biological replicates were employed in each analyzed condition.

### Tissue Sample Preparation for Proteomics Analysis of Differential Cys Alkylation following the FASP Digestion Method

Protein extracts from homogenized tissue were obtained using ceramic beads (MagNa Lyser Green Beads apparatus, Roche, Germany) in extraction buffer (50 mM Tris–HCl, 1 mM EDTA, 1.5% SDS, and 50 mM iodoacetamide (IAM), pH 8.5). Protein concentration was quantified using an RC DC Protein Assay (Bio-Rad). 90 μg of protein extract per sample was subjected to tryptic digestion using the FASP protocol according to the previously published method.^7^ Protein digestion was quenched by adding trifluoroacetic acid to 1% final concentration of 1%, and the peptides were desalted on C18 Oasis HLB extraction cartridges (Waters Corporation, Milford, MA, USA) and dried-down.

### Tissue Sample Preparation for Proteomics Analysis of Differential Cys Alkylation Following the PAC Digestion Method

Protein extracts from homogenized tissue were obtained, as described above. To the volume equivalent 90 μg of protein per sample, 100% of acetonitrile was added to a final concentration of 70% of acetonitrile, followed by 10 μL of hydroxyl beads (Resyn Biosciences). Samples were aggregated by two steps of vortexing for 1 min followed by waiting for 10 min. Samples were placed on a magnetic rack, and the supernatant was discarded. Samples were incubated for 30 min at 56 °C with 100 μL of reducing buffer (50 mM Tris (pH 8.5), 10 mM DTT, 2% SDS) mixing at 350 rpm. 233 μL of 100% of acetonitrile was added to the sample, followed by vortexing for 1 min and resting for 10 min. Samples were placed back on a magnetic rack, and the supernatant was discarded. Samples were incubated for 30 min at room temperature with 100 μL of secondary alkylation buffer (20 mM MMTS, 50 mM HEPES 1 mM EDTA, pH 7.5) mixing at 350 rpm. 233 μL of 100% of acetonitrile was added to the sample, followed by vortexing for 1 min and resting for 10 min. Samples were placed back on a magnetic rack, and the supernatant was discarded. Without removing the samples from the magnetic rack, the samples were washed three times with 1 mL of 95% acetonitrile and two times with 1 mL of 70% ethanol. The digestion was performed overnight in 50 μL of 50 mM TEAB in a ratio 1:40 (w/w, trypsin (Promega):protein). Trifluoroacetic acid was added to a final concentration of 1%, and the peptides were desalted on C18 Oasis HLB extraction cartridges (Waters Corporation) and dried-down.

### Labeling of Peptides Using TMT Reagents Followed by Off-Line HpH Fractionation

Peptides were resuspended in 40 μL of 100 mM TEAB, and peptide concentration was quantified by a Direct Detect IR spectrometer (Millipore, Billerica, MA, USA). Peptides were subjected to multiplexed isobaric labeling using two tandem mass tag 10plex (TMT10plex) kits following manufacturer instructions. Each TMT 10plex batch had one channel reserved for reference internal standard samples created by pooling the four samples. Labeling scheme for TMT #1 was as follows: 126: Pool, 127N to 128N: Baseline, 128 C to 129 C: 120 min ischemia/reperfusion; 130 N to 131 N: 120 min ischemia. Labeling scheme for TMT #2 was as follows: 126: Pool, 127 N to 128 N: Baseline, 128 C to 129 C: 24 h of ischemia/reperfusion; 130 N to 131 N: preconditioning followed by 24 h of ischemia/reperfusion. TMT samples were desalted on C18 Oasis HLB extraction cartridges (Waters Corporation). Desalted peptides were dried down and resuspended in 400 μL of 0.1% TFA, where 40 μL was used for single-shot analysis of the unfractionated sample, and the rest was fractionated into five fractions by high pH reversed-phase chromatography (kit by Thermo Scientific).

### LC–MS/MS Analysis

Samples were analyzed using an Endurance Evosep column (15 cm × 150 μm to C18 1.6 μm) interfaced with the Orbitrap Eclipse Tribrid mass spectrometer (Thermo Scientific) using an EasySpray Ion Source heated to 55 °C. In all samples, spray voltage was set to 2.1 kV, funnel RF level at 40, and heated capillary temperature at 310 °C. Samples were separated on an Evosep One using the gradient for 15 samples per day (SPD).

For DIA analysis, full MS resolution was set to 120,000 at *m*/*z* 200, and full MS AGC target was 300% with a maximum injection time (IT) of 45 ms. The AGC target value for fragment spectra was set to 1000%. 50 windows of 12 Th scanning from 400 to 100 *m*/*z* were employed with an overlap of 1 Da. MS2 resolution was set to 30,000, IT to 54 ms, and normalized collision energy (NCE) to 30%.

For TMT samples, the MS was operated at a full MS resolution of 120,000 with a full scan range of 375–1500 *m*/*z*. The full scan AGC target was set to 100%. During a fixed cycle time of 2 s, precursors were isolated with a window of 1.2 Th and fragmented using HCD with 36% NCE. Fragment ion scans were recorded at 30,000 resolution and with a maximum IT of 54 ms and an AGC target value of 100%.

For comprehensive analysis of DDA samples, LC–MS analysis was done using an Easy nanoLC 1000 (ThermoFisher Scientific) coupled to an Orbitrap Fusion Tribid mass spectrometer (Thermo Fisher Scientific) using an Acclaim PepMap 100 C18 2 cm × 75 μm ID as trapping column (Thermo Fisher Scientific) and a PepMap RSLC C18 EASY-Spray column 50 cm × 75 μm ID as analytical column (Thermo Fisher Scientific) using a 270 min gradient. Mass spectra were acquired with an automatic switch between MS and MS/MS with a “Top-15” method and 40 s of dynamic exclusion. MS spectra were acquired in the Orbitrap analyzer using full ion-scan mode with a 390–1700 *m*/*z* range and 60,000 resolution. The automatic gain control target was set at 1 × 10^6^ with a 50 ms maximum injection time. HCD fragmentation was performed at 30% of normalized collision energy, and MS/MS spectra were analyzed at a 30,000 resolution in the Orbitrap with 100 ms maximum injection time.

### DDA-Based Library Generation

DDA raw files were analyzed using MSFragger (v4.1) and FragPipe (v22) using the Sus Scrofa proteome (UP000008227, accessed 25-03-2024). Variable modifications used were oxidation on methionine, carbamidomethylation and beta-methylthiolation on cysteine, and acetylation on protein N-terminal. The enzyme/cleavage rule was set to trypsin, and a maximum of 3 variable modifications per peptide were allowed. MSBooster was enabled. Postanalysis validation and FDR control were performed using Percolator, while protein inference was performed using ProteinProphet with default parameters in FragPipe. Spectral library generation was enabled with default values. The resulting library file was employed for DDA-based library searches in DIA-NN.

### Peptide and Protein Identification of DIA Data Sets

DIA raw files were analyzed using DIA-NN (v 1.8.1) using an in silico predicted library from the Sus Scrofa proteome (UP000008227, accessed 25-03-2024). For library prediction, the enzyme/cleavage rule was set to trypsin; one missed cleavage per peptide and two variable modifications per peptide were allowed. Variable modifications used carbamidomethylation and beta-methylthiolation on cisteine and oxidation on methionine. MMTS retention times were corrected based on experimental data as follows: iRTs from the DIA-NN spectral library for MMTS-containing peptides were corrected using the following eq 1

1 $$corrected\;iRT=0.86^{*}original\;iRT+27$$

Cross-run normalization was turned-off, and remaining options were set as default.

If not indicated in relevant figures, peptides carrying cysteines, one labeled with IAM and the other with MMTs were not accounted.

### Peptide and Protein Identification of TMT Data Sets

TMT raw files were analyzed using MSFragger (v4.1) in FragPipe (v22) using the Sus Scrofa proteome (UP000008227, accessed 25-03-2024). Variable modifications used were oxidation on methionine, carbamidomethylation and beta-methylthiolation on cysteine, and acetylation on protein N-terminal. TMT10plex tag was set as fixed modification in peptide N-terminus and lysine, as well as variable modification in serine. The enzyme/cleavage rule was set to trypsin, and a maximum of 3 variable modifications per peptide were allowed. MSBooster was enabled. Post-analysis validation and FDR control were performed using Percolator, while protein inference was performed using ProteinProphet with default parameters in FragPipe. TMT reporter intensities were extracted directly from the mzml converted raw files using an in-house plugin implemented in the iSanXoT (v.1.2.13) software in which isotopic contamination correction was also performed.

If not indicated in relevant figures, doubly modified peptides (i.e., peptides carrying two cysteine one labeled with IAM and another with MMTs) were not accounted.

### Peptide and Protein Quantification

The quantitative information derived from TMT reporter intensities and DIA precursor intensities were integrated from the spectrum level (for TMT data) or precursor (for DIA data) to the peptide level (“pr2p”) and subsequently to the protein level (“p2q”), according to the WSPP model^20^ and the generic integration algorithm (GIA),^21^ using the iSanXoT program version 1.2.13 ^22,23^ . In this model, quantitative protein values are expressed using the standardized variable Z (i.e., normalized log2-ratios expressed in units of standard deviation according to the estimated variances).

Zp2q values were employed to plot sigmoid curves and heatmaps throughout the publication. Sigmoid curves were generated by grouping the peptides into three categories: reduced cysteine peptides (labeled with IAM), reversibly oxidized cysteine peptides (labeled with MMTS), and other peptides (noncysteine-containing peptides). Peptides were ranked according to their Zp2q values, and for each peptide, we divided their rank by the total number of peptides in their category (N). Sigmoid curves were therefore generated by plotting rank/N versus Zp2q.

### Library-Free DIA Software Benchmark

For the comparison between DIA search engines, two raw files from porcine atrial tissue were analyzed using DIA-NN v2.0, Spectronaut v19.2, and AlphaDIA v1.10.3. In all three cases, the Sus scrofa FASTA reference proteome from UniProt (UP000008227 January 2025 release, containing 46,243 proteins) was used as the search database.

Prior to DIA-NN data analysis, a spectral library was generated from the FASTA file, using the following parameters: --min-pep-len 7, --max-pep-len 30, --min-pr-mz 300, --max-pr-mz 1800, --min-pr-charge 1, --max-pr-charge 4, --cut K*,R* (use of trypsin as the digestion enzyme), --missed-cleavages 1, --var.-mods 2, --smart-profiling, --var.-mod UniMod:39,45.987721,C, --var.-mod UniMod:4,57.021464,C, --var.-mod UniMod:35,15.994915,M and --full-unimod. The resulting speclib file was converted to Parquet format using DIA-NN, and subsequently, the retention times of precursors containing UniMod:39 were corrected using an R script, following the equation: corrected iRT = 0.86 × Original iRT +27. Finally, the corrected library was used to perform a search in DIA-NN with the following parameters: qvalue 0.01, --gen-spec-lib, --var.-mods 2, --reanalyse, --smart-profiling, --no-norm, and --full-unimod.

The search with Spectronaut was performed using the directDIA+ mode, specifying Trypsin/P as the digestion enzyme for the FASTA, allowing 1 missed cleavage, enabling N-terminal methionine removal, and setting the peptide length between 7 and 30 amino acids. The maximum number of variable modifications was set to 2, considering oxidation of methionine and carbamidomethylation and methylthiolation in cysteine as variable modifications. No fixed modifications were specified. An FDR threshold of 0.01 was set for all levels (PSM, Peptide, and Protein Group). All quality filters were set as a default.

In the analysis with AlphaDIA, the transfer learning and Match between Runs steps were enabled, and trypsin was specified as the digestion enzyme with a maximum of 1 missed cleavage. Precursor lengths were set between 7 and 30 amino acids, their *m*/*z* values were set between 300 and 1800, and possible charge states were set between 1 and 4. The *m*/*z* range for fragment ions was limited between 200 and 1800. The maximum number of variable modifications was set to 2, including oxidation of methionine and carbamidomethylation and methylthiolation on cysteine. No fixed modifications were specified. The FDR value was set to 0.01.

The comparison of precursors identified by the different tools was performed using the files report.pr_matrix.tsv and report.tsv from DIA-NN, Report_BGS Factory Report (Normal.tsv) from Spectronaut, and precursors.tsv from AlphaDIA.

## Results and Discussion

### Protein Aggregation Capture-Based Differential Alkylation for REDOX (PACREDOX) Proteomics

In this work, we propose an adaptation of the PAC digestion protocol^15^ that supports differential alkylation of Cys, named PACREDOX. The method incorporates three additional steps into the standard PAC sample preparation workflow (Figure 1A). First, samples are homogenized and lysed, and proteins are denatured in an SDS-containing buffer in the presence of iodoacetamide (IAM), which is used to carbamidomethylate free thiols. After being boiled, the samples are aggregated onto the hydroxyl beads by adding acetonitrile. After buffer removal, the samples are reduced in a buffer containing dithiothreitol (DTT). Following reduction, the aggregation step is repeated with acetonitrile, and the buffer is removed again. The freshly created free thiols (corresponding to reversibly oxidized Cys) are then alkylated by incubating the beads with a buffer containing methylmethanethiosulfonate (MMTS). After the second alkylation, the proteins are aggregated once more onto the beads by using acetonitrile, and the PAC protocol proceeds without further alterations. Importantly, we found that the two additional aggregation steps are necessary, since skipping them after the reduction or after the second alkylation step resulted in significant protein losses (Supplementary Figure S1A).

**Figure 1:**
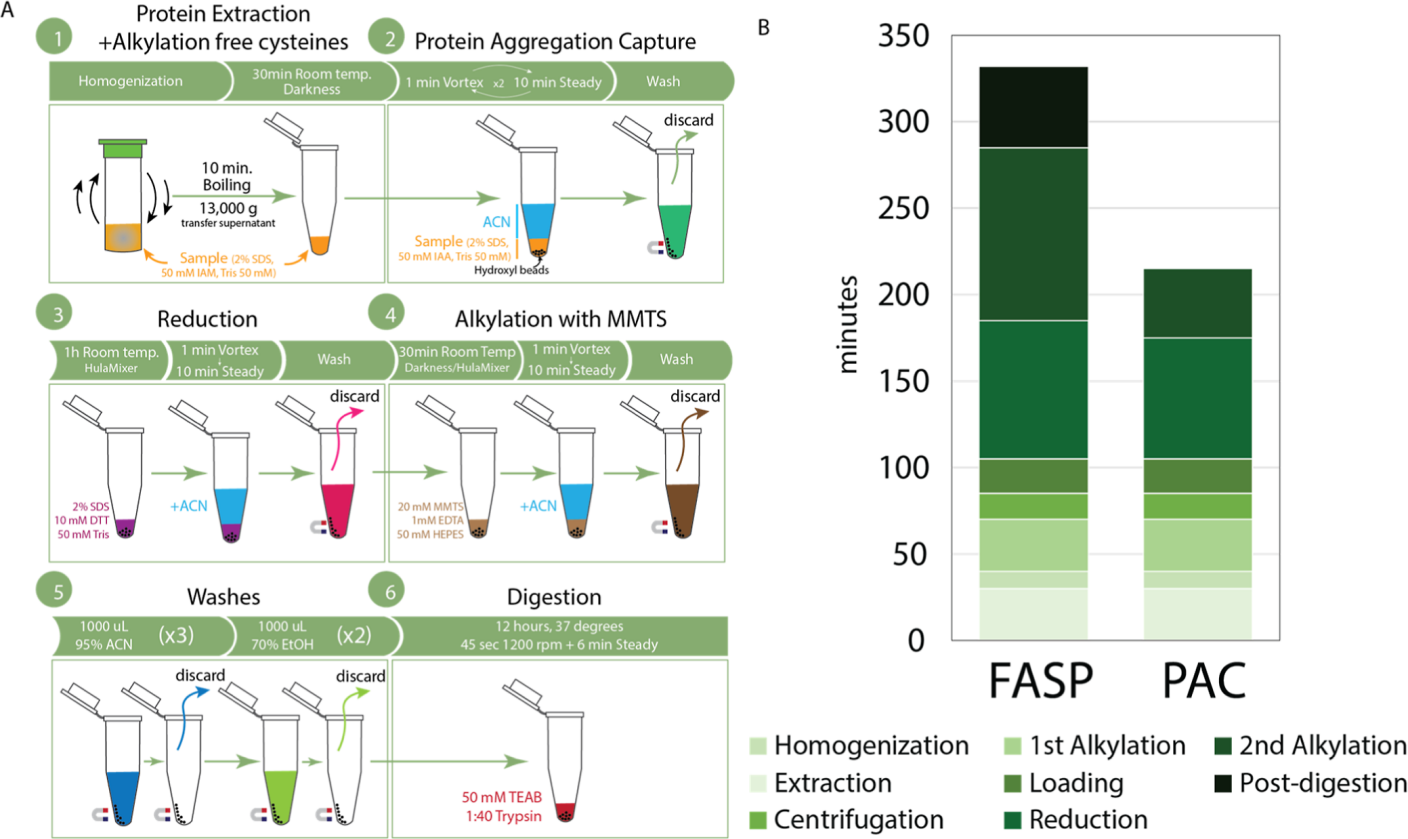
(A) PAC adapted workflow for differential alkylation of Cys thiol groups for redox proteomics. (B) Stacked bar plot of expected times required for each step in FASP or PAC-based protocols for redox proteomics.

We compared this new protocol against the FASP-based differential alkylation protocol (FASILOX) previously developed by our group.^7^ The FASILOX protocol is based on the same differential alkylation strategy with IAM and MMTS, but it was originally designed to incorporate isobaric labeling for quantification. To assess the advantages of our PAC-based approach, we first quantified the time required for differential alkylation and digestion when using PAC as described here or FASP as outlined in the FASILOX protocol (Figure 1B). The centrifugation, washing, and postdigestion steps in the FASILOX method increase sample preparation time compared to the equivalent steps in PACREDOX. Additionally, it is important to note that this time estimate does not account for concomitant problems such as potential blockages of the FASP filters.

### Optimization of “in Silico” Libraries for DIANN Analysis of Redox Samples

To validate the usability of the PACREDOX protocol, we analyzed whether the PAC-based differential alkylation is compatible with various quantification strategies. First, we analyzed protein extracts from a porcine model for atrial fibrillation (AF) subjected to PACREDOX or FASP-based preparations (as in FASILOX) and analyzed using DDA. We observed that both PAC and FASP preparations resulted in equivalent depth of nonmodified, IAM-labeled and MMTS-labeled peptides (Supplementary Figure S1B). Both methods allowed us to estimate that the proportion of MMTS-labeled (oxidized) peptides with respect to IAM-labeled (reduced) peptides was around 30%, showing that the PAC-based protocol does not introduce any significant bias in the quantification of differential redox Cys species.

Next, we evaluated whether PACREDOX could be used to study the redox proteome through label-free quantification and DIA analysis. Until now redox proteomics has been performed almost exclusively using DDA approaches due to the lack of in silico spectral libraries that encode peptides modified with secondary alkylation compounds, such as MMTS. Indeed, previous redox proteomics studies using DIA (9) have relied on experimentally generated libraries.

We evaluated the similarity between the fragmentation profiles of peptides labeled with IAM (i.e., carbamidomethylated peptides) and those labeled with MMTS (i.e., methylthiolated peptides). For this purpose, we analyzed the PACREDOX DDA data and compared the fragmentation profiles of IAM-labeled against their MMTS-labeled counterpart peptides by measuring the correlation between the intensity of the equivalent fragments (Figure 2A). We found that the correlation of fragmentation profiles of the ∼400 peptide pairs analyzed showed a median Pearson correlation value of 0.91, which increased when more than 12 fragments were available (Figure 2B). This analysis thus showed that MMTS-labeling does not appreciably alter the fragmentation profile compared with IAM-labeling indicating that *in silico* fragmentation predictions of IAM-labeled peptides could be translated to MMTS-labeled peptides. To test this possibility, we included peptide fragmentation spectra of MMTS-labeled peptides to the *in silico* library generated by DIA-NN which already contained IAM-labeled peptides. Using this extended library, the proportion of identified MMTS-labeled peptides with respect to IAM-labeled peptides was only 7% (Figure 2F), in contrast to using an experimental DDA-based library, with which the proportion of MMTS-labeled peptides was 30% (Figure 2F), in agreement with the results obtained by direct DDA analysis.

**Figure 2:**
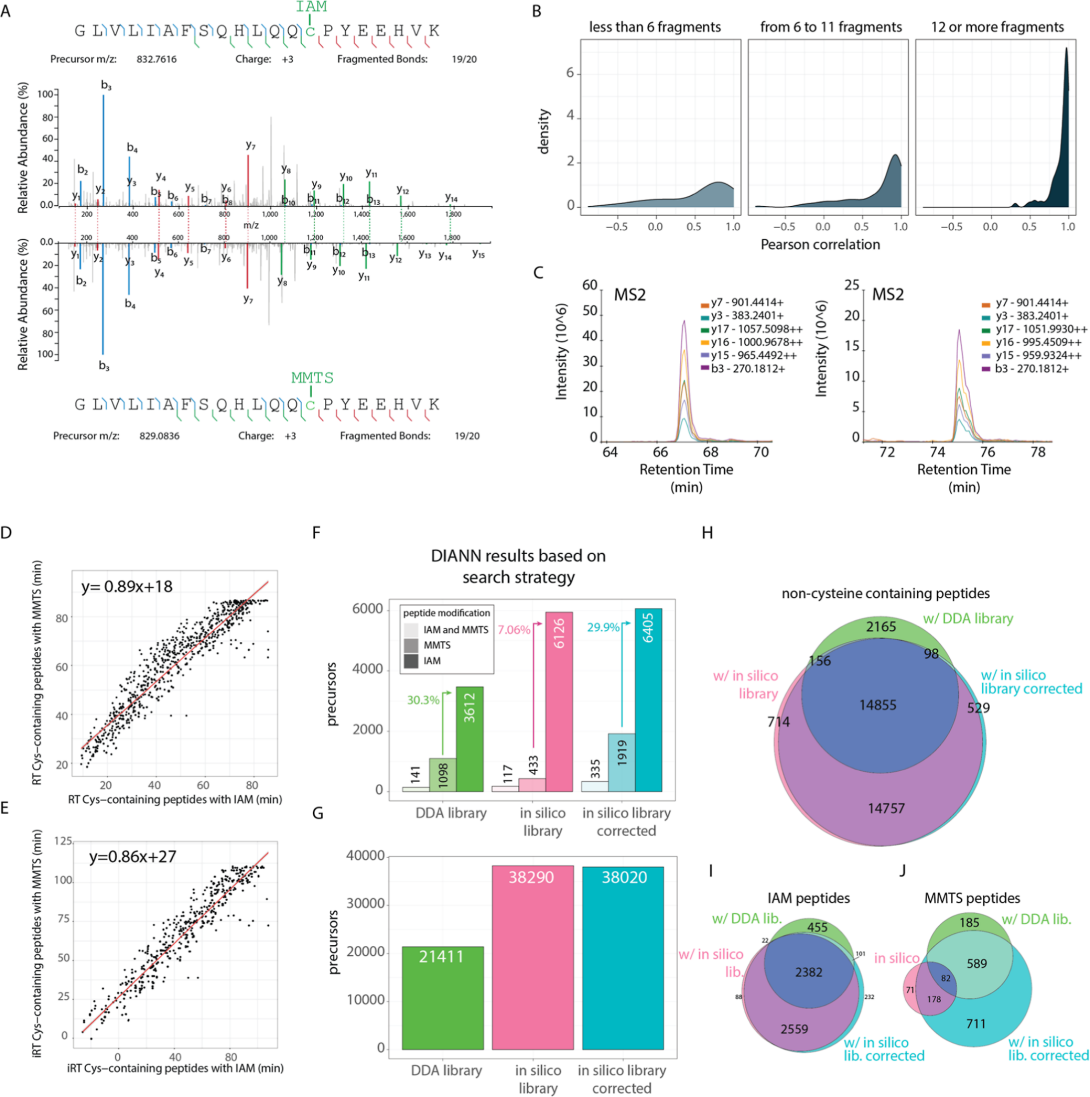
(A) Fragmentation spectrum of a Cys-containing peptide labeled either with IAM (top) or with MMTS (mirrored, bottom). In blue: b-series fragments; in red: common y-series fragments and in green: specific y-series fragments. (B) Density plots of Pearson correlation values calculated between the fragmentation profiles (of equivalent fragment ion series) of IAM-labeled peptides and their MMTS-labeled counterparts, based on the number of fragments available for the comparison. (C) Extracted ion chromatograms at MS2 level for the two peptides from panel A (IAM-labeled on the left, MMTS-labeled on the right). (D) Scatter plot of representing retention times (RT) from IAM-labeled peptides vs their MMTS-labeled counterparts in an 88 min LC-run. Red line corresponds to the regression line, which equation is indicated in the top left corner. (E) Scatter plot representing iRTs from DIA-NN transformed library from IAM-labeled peptides vs their MMTS-labeled counterparts in the same analysis as in panel D. Red line corresponds to the regression line, which equation is indicated in the top left corner. (F,G) Bar plot of the number of Cys-labeled precursors (F) or non-Cys-containing precursors (G) identified in two samples in DIA-NN with three different search strategies: using a library generated in FragPipe from DDA files (green), using an *in silico* library from DIA-NN (pink) and using an “in silico” library from DIA-NN in which the retention times from MMTS-labeled peptides was corrected according to the equation from panel E (blue). (H–J) Euler diagrams showing the overlap between identified precursors in DIA-NN searches, either non-Cys containing ones (H), IAM-labeled (I) or MMTS-labeled (J), when using either DDA-based library, DIA-NN *in silico* library or DIA-NN *in silico* library corrected for retention time.

Further examination of the results obtained using the DDA-experimental library revealed that MMTS-labeled peptides eluted consistently later than their IAM-labeled counterparts (Figure 2C,D), presumably due to their higher hydrophobicity. We noticed that the iRTs of MMTS-labeled peptides could be inferred by a linear transformation of the iRT values of the IAM-labeled counterparts from DIA-NN libraries (Figure 2D,E). Therefore, we used this transformation to adjust the predicted retention times of MMTS-labeled peptides in the extended *in silico* library and reanalyzed the samples using DIA-NN. Encouragingly, the iRT correction allowed us to recover the expected rate of reversibly oxidized to reduced Cys (30%) (Figure 2F and Supplementary Table S1), significantly enhancing the overall DDA coverage of the thiol redox landscape without adversely affecting the identification of other peptides (Figure 2G–J).

Furthermore, we explored other search engines, such as Spectronaut or AlphaDIA,^24^ which could potentially allow for differential alkylation analysis of DIA data. On the one hand, Spectronaut deconvolutes DIA spectra to generate pseudo-DDA data that can be analyzed using spectrum-centric search strategies. AlphaDIA, on the other hand, uses an algorithm based on library prediction, in which different modifications can be trained. Analysis of our samples in Spectronaut (v19) recapitulated the proportion of 30% oxidized to reduced Cys expected in our model (Supplementary Figure S2A,B and Supplementary Table S2). AlphaDIA (v1.10.3) resulted in slightly fewer identifications than Spectronaut or DIA-NN, but it still recapitulated the correct proportion of oxidized to reduced Cys (Supplementary Figure S2A). However, we found a lack of reproducibility on the MMTS-labeled peptides among search engines (Supplementary Figure S2B). When analyzing the retention time relationship between the identified IAM- and MMTS-labeled peptides, we observed two distributions in AlphaDIA and Spectronaut: one that followed the same trend as reported experimentally and one distribution in which MMTS-labeled peptides had the same retention time as their IAM-labeled counterparts (Supplementary Figure S2C). We hypothesized that a significant majority of those MMTS-labeled peptides could have been incorrectly identified by the fragments from the IAM-labeled peptide, since their masses fell within the fragmentation window of their IAM-labeled counterpart (Supplementary Figure S2D) given the isolation width used for this DIA analysis (12 Da). Moreover, since fragments belonging to y-series tend to predominate, it might have limited the detection of diagnostic fragments for the labeled cysteine of peptides in which the cysteine is close to the N-termini (Supplementary Figure S2E–K). Overall, this benchmark reflects the importance of correct retention time assignation for proper peptidoform identification to avoid false positives in cases in which, due to the isolation width used, different peptidoforms can be potentially cofragmented.

### PACREDOX Using DIA Recapitulates the Oxidative Patterns Produced by Ischemia-Reperfusion Injury

After demonstrating that PAC-based sample preparation can be combined with DIA to analyze the thiol redox proteome, we benchmarked this approach in a biological model of oxidative stress. We used myocardial tissue protein extracts from a pig model of ischemia-reperfusion (I/R)^25^ previously analyzed using FASILOX^12^ to assess the oxidative damage produced by reperfusion and to evaluate the cardioprotective effect of preconditioning.^26,27^ We compared samples from three pigs with no intervention in baseline conditions to those from three pigs subjected either to 40 min of ischemia followed by 90 min of reperfusion (120 min R), or from three pigs subjected to 120 min of ischemia without later reperfusion (120 min NOR). Additionally, we compared samples from three pigs subjected to ischemic preconditioning followed by 24 h reperfusion (PRE 24 h) and those from three nonpreconditioned animals at 24 h of reperfusion (Figure 3A).

**Figure 3:**
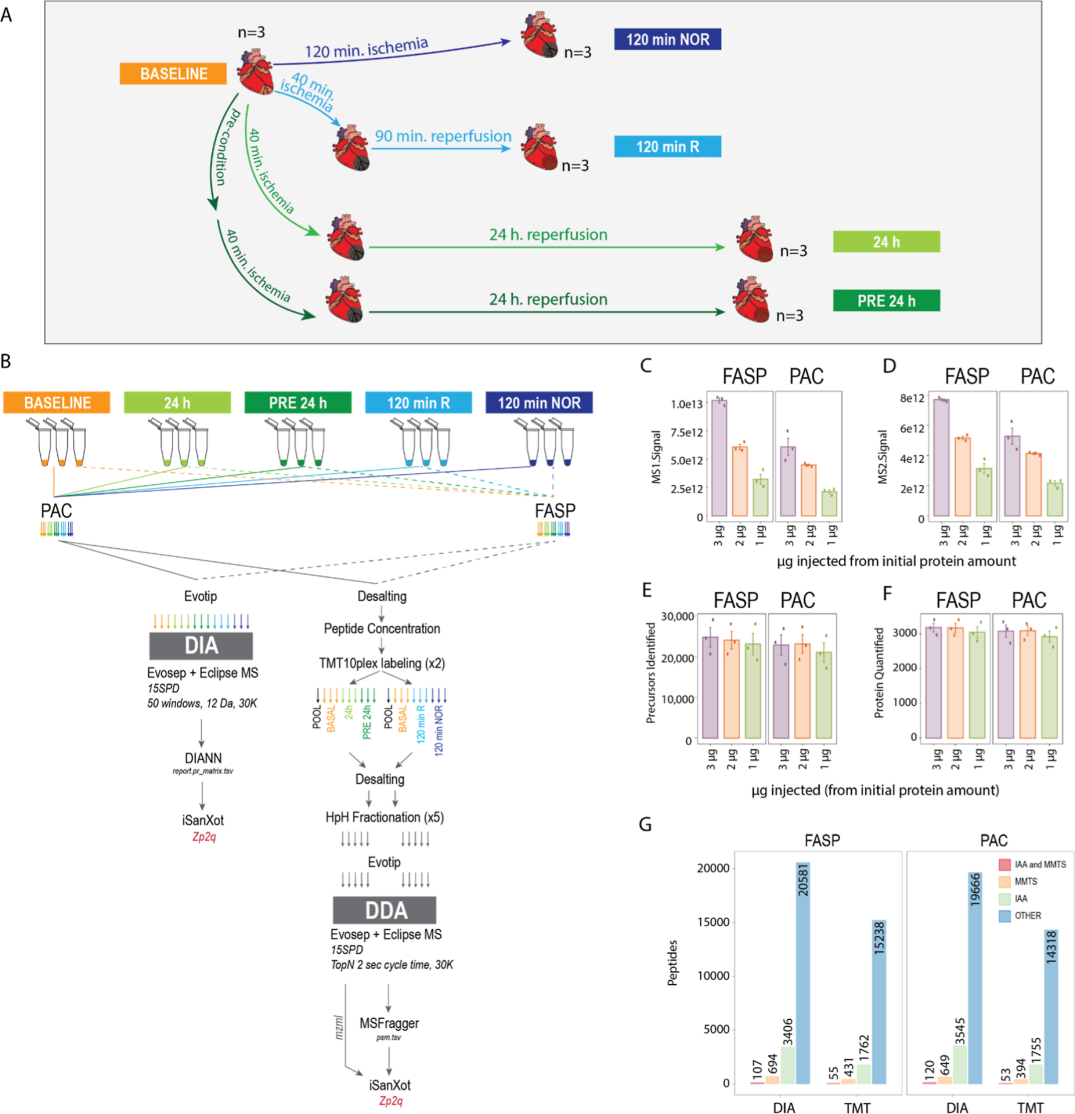
(A) Experimental design to study the effect of ischemia/reperfusion injury on cardiac tissue. (B) Experimental workflow either using PAC or FASP-based digestion followed by DIA or TMT quantification. (C,D) MS1 (C) and MS2 (D) signal measured in DIA analysis injecting different expected amounts based on starting protein quantities (assuming 100% recovery from digestion) in either FASP or PAC protocols. (E,F) Number of precursors (E) and proteins (F) identified using DIA analysis when injecting different expected amounts based on starting protein quantities in either FASP or PAC protocols. In C to F panels: the bars represent the mean ± SEM of 3 biological replicates. (G) Number of peptides used for quantification in PAC or FASP and DIA or TMT workflows. Blue: non-Cys containing peptides; green: Cys-carbamidomethylated peptides; orange: Cys-methylthiolated peptides; and red: peptides containing both carbamidomethylated and methylthiolated peptides.

For a reliable benchmarking, we reprocessed the same protein extracts used by Binek et al.,^25^ analyzing the same sample amounts by either PAC- or FASP-based digestion protocols, followed by quantification using either TMT-labeling or label-free DIA analysis. For this comparison, we chose an experimental design (Figure 3B) that reasonably balanced sample preparation and MS analysis times. Sample preparation using the label-free protocol was completed within 2 days, while the TMT-labeling protocol required additional time for desalting, peptide labeling, and offline peptide fractionation (Figure 3B). On the other hand, the label-free MS data acquisition was completed in 24 h using a 15 SPD gradient on an Evosep One (15 runs, 1.6 h of MS time per sample), while the labeled samples required slightly less time (12 runs taking 19.2 h, 1.3 h per sample).

To evaluate the performance of each protocol, we analyzed the samples of each digestion strategy loading different proportions of the protein digest into Evotips. Comparing the results obtained from DIA analysis, we observed that FASP consistently yielded higher MS1 and MS2 signal intensities than PAC (Figure 3C,D), indicating that the FASP protocol had fewer peptide losses (Figure 3C,D). Despite this, protein and precursor identification performance did not significantly differ between PAC and FASP samples (Figure 3E,F). When comparing all sample preparation and MS data acquisition methods specifically at the peptide level, we found that label-free DIA had a consistently higher identification rate than did TMT (Figure 3G). Overall, these results support the validity of PACREDOX combined with DIA as an attractive alternative to more elaborated protocols such as FASILOX.

We next assessed the quantification performance for PAC and FASP-based protocols coupled to either label-free DIA analysis or the original FASILOX workflow (i.e., FASP coupled to isobaric labeling). The quantitative data at the scan (for TMT) or precursor (for DIA) levels were processed by the iSanXoT package,^22,23^ using the standard workflow for the analysis of post-translational modifications.^23^ The quantitative values were expressed as log_2_-ratios, using as a reference for the denominator the average of the baseline condition samples. The quantitative scan (for TMT) or precursor (for DIA) values were then integrated to peptide values (pr2p), and the peptide values to protein values (p2q), to obtain standardized peptide values *Zp2q*, which are independent from protein changes.^23^ iSanXoT uses the generic integration algorithm (GIA)^20^ to model the distribution of variances in each integration. We observed that the global variance at peptide (p2q) and protein (q2all) was clearly higher in DIA data than that of TMT irrespective of the sample preparation protocol used (Supplementary Figure S3A,B). This agrees with the lower accuracy of label-free quantification in comparison with stable isotope labeling quantification. The better precision in TMT data is linked to the fact that the intensities of the different samples are measured within the same spectrum, while in DIA, each sample is processed in different chromatographic runs.

In order to quantify the differential thiol redox proteome, we analyzed the distribution of peptide values for three separate populations: reversibly oxidized Cys-containing peptides, reduced Cys-containing peptides, and non-Cys-containing peptides. The *Zp2q* values from the peptides without Cys and the reduced Cys-peptides followed the theoretical N­(0,1) distribution (Figure 4A–C) both for FASILOX and for the PACREDOX-DIA approach, demonstrating that the results obtained with the PACREDOX protocol, like those with FASILOX,^7^ can be modeled using the statistical GIA framework. In contrast, the distribution of oxidized Cys-containing peptides deviated from the null hypothesis, showing increased levels in the samples subjected to ischemia-reperfusion for 120 min and 24 h (Figure 4A–C) both in FASILOX and PAC-DIA data, reflecting oxidative thiol damage, as previously described.^12^ Notably, in the absence of reperfusion, the oxidative damage after 120 min was clearly lower than that observed with 90 min reperfusion using both preparation protocols (Figure 4A,B,E and Supplementary Figure S3C). Similarly, the data obtained by the two approaches also showed that ischemia preconditioning mitigated the oxidation observed after 24 h of I/R (Figure 4C–E and Supplementary Figure S3C). All these results indicate that neither the choice of digestion strategy nor the quantification strategy alter the outcomes of the experiment, which reflect the same biological effects.

**Figure 4:**
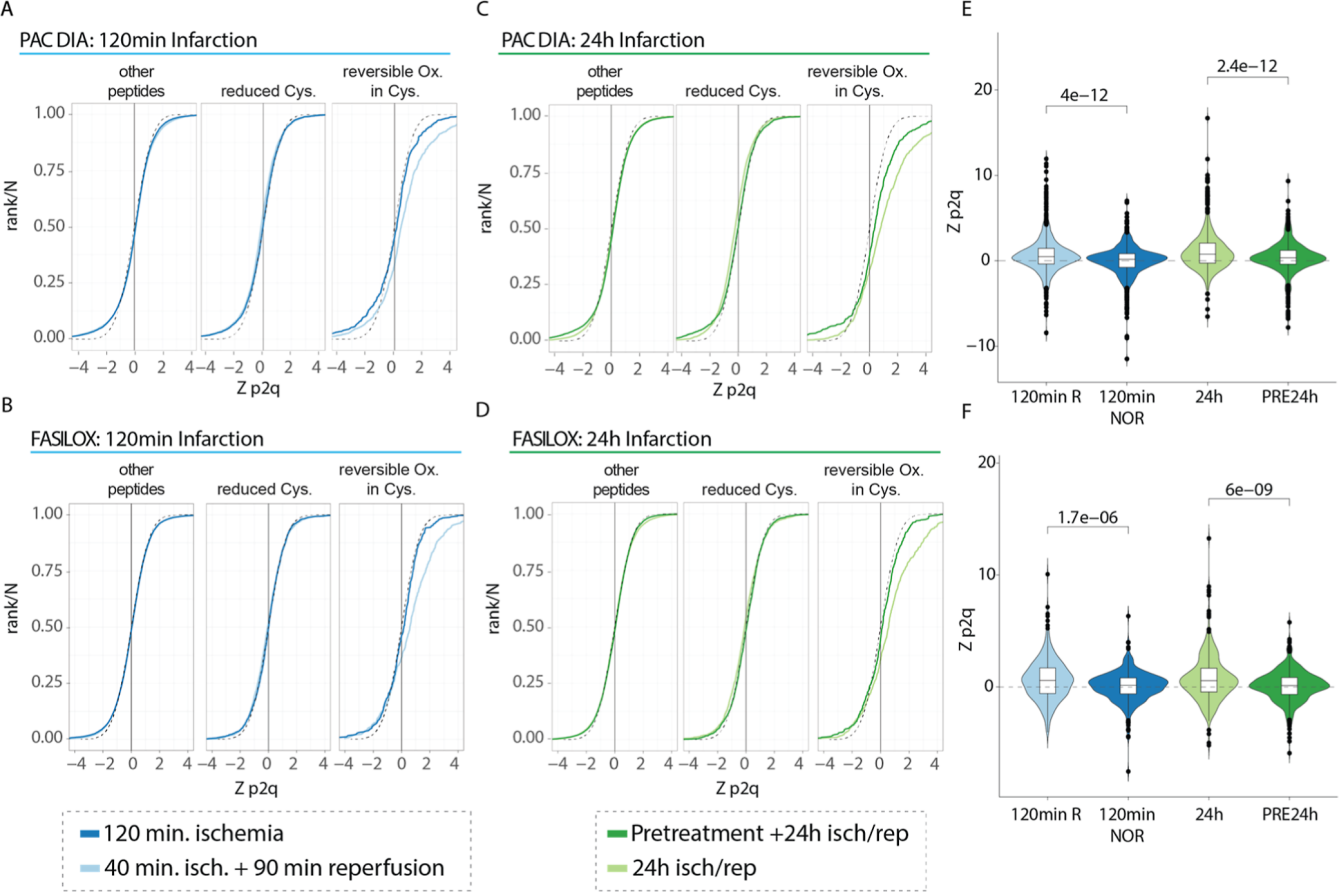
(A–D) Distribution of Z values from the integration peptide to protein (p2q) for three peptide populations: non-Cys containing peptides, reduced Cys and reversibly oxidized Cys: 120 min of I/R experiment analyzed by PACREDOX-DIA (A) or by FASILOX (B). In A,B, light blue corresponds to 40 min ischemia +90 min of reperfusion (120 min R) and dark blue corresponds to 120 min of ischemia without reperfusion (120 min NOR). (C) 24 h of I/R experiment analyzed by PACREDOX-DIA (C) or by FASILOX (D). In (C,D), light green corresponds to 40 min ischemia +24 h of reperfusion (24 h) and dark green corresponds to 40 min ischemia +24 h of reperfusion on preconditioned pigs (PRE24 h). In all panels, the black dotted line represents the normal distribution N­(0,1). (E,F) Boxplots and violin plots of the Zp2q values for MMTS-labeled peptides in the different experiments aalyzed by PAC-DIA (E) or FASILOX (F), all relative to baseline samples. On top, p-values from a two-sample *t*-test.

However, it is relevant to indicate that DIA achieved higher coverage than TMT of the thiol redox proteome. While DIA provided evidence of oxidized disulfide bonds in 177 protein-coding genes (Supplementary Figure S4A), TMT covered only 90 protein-coding genes with reversibly oxidized Cys (Supplementary Figure S5A). While the category enrichment analysis revealed four main functions that grouped most of these proteins in both approaches, namely, cellular respiration, humoral immune response, cell adhesion, and sarcomere organization, the coverage of those pathways was more extensive in DIA derived data (Supplementary Figures S4B and S5B). Moreover, DIA was able to identify proteins that can be relevant in the I/R injury process that were not identified by TMT. Such is the case of Myomesin 2 (MYOM2), which is a major component of the vertebrate myofibrillar M band which has been described to be oxidized in heart failure.^29^ DIA generated exclusive evidence of four reversibly oxidized Cys-containing peptides, which show the expected effect of increased oxidation after I/R injury (Supplementary Figure S3D).

Finally, we assessed technical reproducibility in DIA between PAC and FASP-based workflows. We already showed that both strategies report a similar number of peptides but FASP had better recovery. From a quantification perspective, however, we did not observe relevant differences between both strategies when using DIA (Supplementary Figure S6A,B), and the quantification of reversibly oxidized peptides correlated well between both digestion strategies (Supplementary Figure S6C).

## Conclusions

In this study, we introduced a novel sample preparation strategy leveraging the Particle Aggregation Capture approach for protein digestion, enabling differential alkylation and facilitating an in-depth analysis of the thiol redox proteome. This methodology significantly reduces sample processing time, which is crucial for increasing experimental throughput and potentially minimizing the variability associated with complex preparation techniques. Importantly, in contrast with the FASP method, the PAC approach can be easily implemented into robotized workflows, allowing its automated application to a very large number of samples.

Additionally, we demonstrate that this kind of thiol redox proteomics, originally proposed for isobaric labeling, can be performed using label-free strategies and is compatible with data-independent acquisition (DIA) without requiring experimental libraries. Notably, we utilized *in silico* libraries that can be adjusted to accommodate additional modifications beyond their initial training.

Our findings show that DIA can achieve a deeper proteome coverage compared to data-dependent acquisition (DDA) methods, such as tandem mass tag (TMT) labeling, given similar sample processing times. Note that the exact time required for sample preparation and MS analysis for each of the two strategies will depend on the specific experimental design, including the number of replicates, peptide fractions, and TMT multiplexing. Thus, a depth comparable to DIA might be achievable by using 18-plex TMT and increasing the number of fractions analyzed.^30^ From a quantification standpoint, however, DIA data displayed greater variability than TMT, an important consideration in thiol redox proteomics, where quantification is peptide-based. Previous reports^31^ describe a connection between ratio compression and reduced accuracy, with improved precision in TMT quantification, although these factors were only compared across TMT experiments. In this work, the source of precision divergence between TMT and DIA comes mainly from the acquisition step, where peptide measurements for different samples are performed in the same spectrum for TMT and in separate runs for DIA. As a result, DIA quantifications are affected by variability in chromatographic separation, ionization, and MS acquisition.

This variability must be considered when designing experiments since achieving comparable statistical power with DIA as with TMT may require additional replicates to balance the quantitative error. While a detailed comparison between label-free and label-based methods is outside the scope of this work, we believe that the simplicity, lower cost, and peptide coverage of the DIA-based approach make it an attractive choice for redox proteomics.

Finally, our work shows for the first time that the Generic Integration Algorithm,^20^ applicable through the iSanXoT package,^23^ is a valid model for the accurate analysis of DIA data. Using iSanXoT provides the option of constructing user-defined, fully automated and reusable workflows for systematic analysis of specific populations of Cys peptides using a robust and validated statistical model for quantitative analysis of post-translational modifications.

In summary, redox proteomics, driven by advances in mass spectrometry and computational tools, offers powerful methodologies for understanding redox PTMs on Cys residues. Through a combination of differential alkylation, enrichment strategies, and evolving MS techniques, redox proteomics continues to describe the complex redox landscape in cellular systems. While challenges remain, particularly regarding library-free approaches and tool accessibility, ongoing innovations such as the one presented in this work promise to expand our understanding of protein modifications and their impact on cellular function.

## Supplementary Material





## Data Availability

The mass spectrometry proteomics data have been deposited to the ProteomeXchange Consortium via the PRIDE^1,32,33^ partner repository with the data set identifier PXD060030.

## Notes

Software benchmarking and biological interpretation of the data, software comparison for DIA PACREDOX analysis, quantification benchmark FASILOX vs DIA-PACREDOX,STRING analysis of DIA PACREDOX analysis of ischemia-reperfusion injury, string analysis of FASILOX analysis of ischemia-reperfusion injury, and comparison of PAC against FASP for DIA REDOX analysis (Figures S1 to S6) (PDF)
Excel document containing identification and quantification results of the data sets for method optimization and ischemia-reperfusion analysis (Tables S1 to S4) (XLSX)
