# Supplementary material for: Benchmark for Quantitative Global and Redox Proteomics Analysis by Combining Protein-Aggregation Capture and Data Independent Acquisition
Source: Anal Chem. 2025 Sep 3;97(36):19730–41. doi: 10.1021/acs.analchem.5c03294 (PMC12445338; doi:10.1021/acs.analchem.5c03294)
Supplement: Supplementary file 1 [file ac5c03294_si_001.pdf]

Supporting information:

Benchmark for quantitative global and redox proteomics analysis by combining Protein-Aggregation Capture and Data Independent Acquisition

Ana Martinez-Val[1,2*], Samuel Lozano-Juárez[1], Jorge Lumbreras Burgueño[9], Irene Rodríguez[9], Marinela Couselo-Seijas[3], Ana Simón-Chica[3], Carlos Galán-Arriola[2,4], Rodrigo Fernández-Jiménez[2,5,6,7], Estefanía Núñez[1,2], Inmaculada Jorge[1,2], David Filgueiras-Rama[2,3,6,7], Borja Ibáñez[2,4,8], Jesús Vázquez[1,2*]

[1] Cardiovascular Proteomics Laboratory, Centro Nacional de Investigaciones Cardiovasculares Carlos III (CNIC), 28029, Madrid, Spain
[2] Centro de Investigación Biomédica en Red de Enfermedades Cardiovasculares (CIBERCV), 28029, Madrid, Spain
[3] Centro Nacional de Investigaciones Cardiovasculares Carlos III (CNIC), Novel Arrhythmogenic Mechanisms Program, 28029, Madrid, Spain
[4] Translational Laboratory for Cardiovascular Imaging and Therapy, Centro Nacional de Investigaciones Cardiovasculares Carlos III (CNIC), 28029, Madrid, Spain
[5] Cardiovascular Health and Imaging Laboratory, Centro Nacional de Investigaciones Cardiovasculares (CNIC), 28029, Madrid, Spain
[6] Instituto de Investigación Sanitaria del Hospital Clínico San Carlos (IdISSC), Cardiology Department, 28040, Madrid, Spain
[7] Department of Cardiology, Hospital Universitario Clínico San Carlos (IdISSC), 28040, Madrid, Spain
[8] IIS-Fundación Jiménez Díaz Hospital, 28040, Madrid, Spain
[9] Proteomics Unit, Centro Nacional de Investigaciones Cardiovasculares Carlos III (CNIC), Madrid 28029, Spain.

*Correspondence to: ana.martinezdelval@cnic.es and jesus.vazquez@cnic.es

Table of Contents

	Page
Method optimization and benchmark	S2
Software comparison for DIA PACREDOX analysis	S3
Quantification benchmark FASILOX vs DIA-PACREDOX	S5
String analysis of DIA PAC REDOX analysis of ischemia-reperfusion injury	S6
String analysis of FASILOX analysis of ischemia-reperfusion injury	S7
Comparison of PAC against FASP for DIA REDOX analysis	S8
References	S8

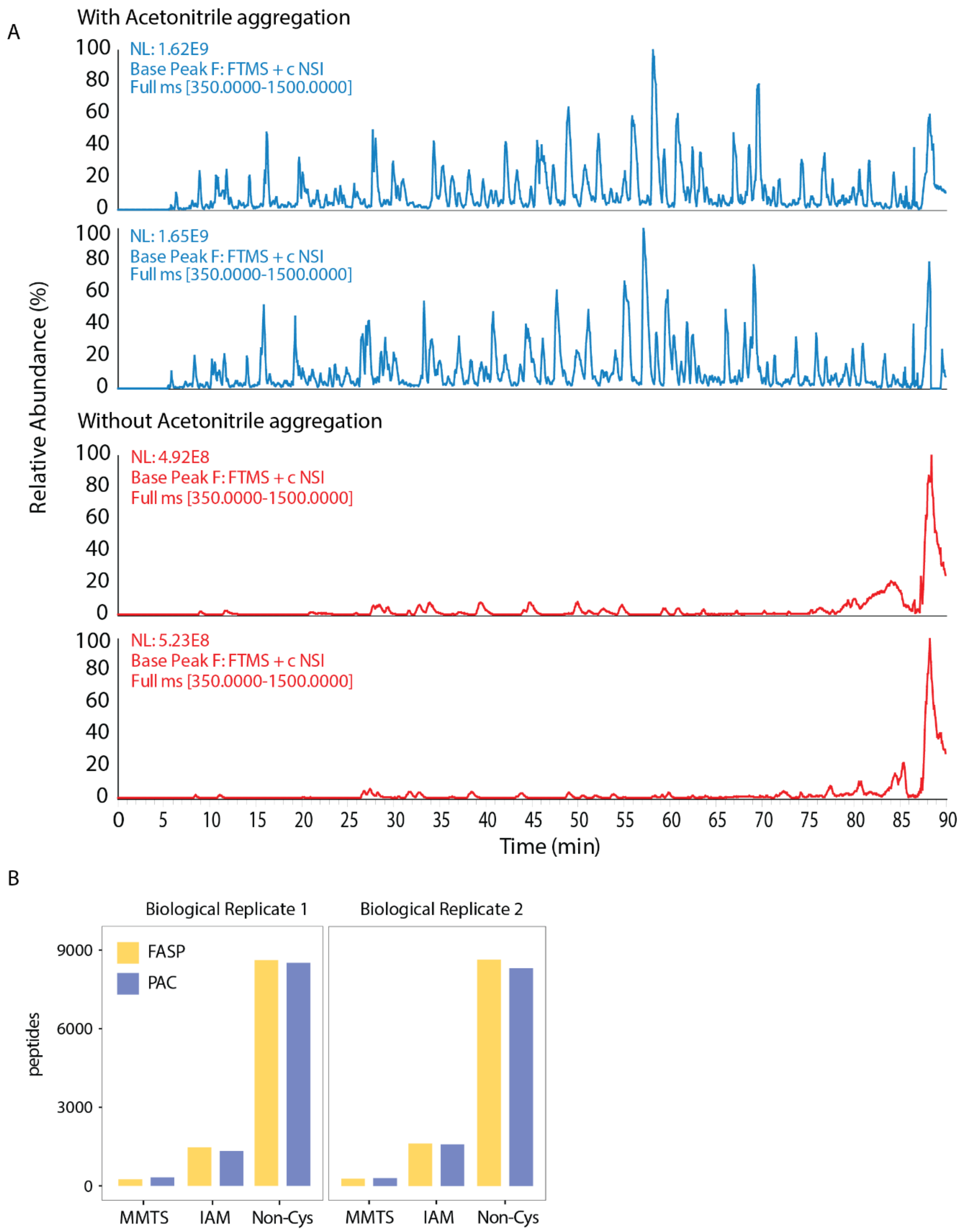

Supplementary Figure S1. *(A) Chromatograms from LC-MSMS analysis of samples generated using PAC-REDOX in which aggregation was used between each derivatization step (top, n=2 replicates) or in which no aggregation was performed between derivatization steps (bottom, n=2 replicates). (B) Number of identified peptides in two raw files (biological replicates) from porcine atrial tissue processed using the PACREDOX protocol (purple) or using FASP (yellow), and analysed using DDA in MSFragger.*

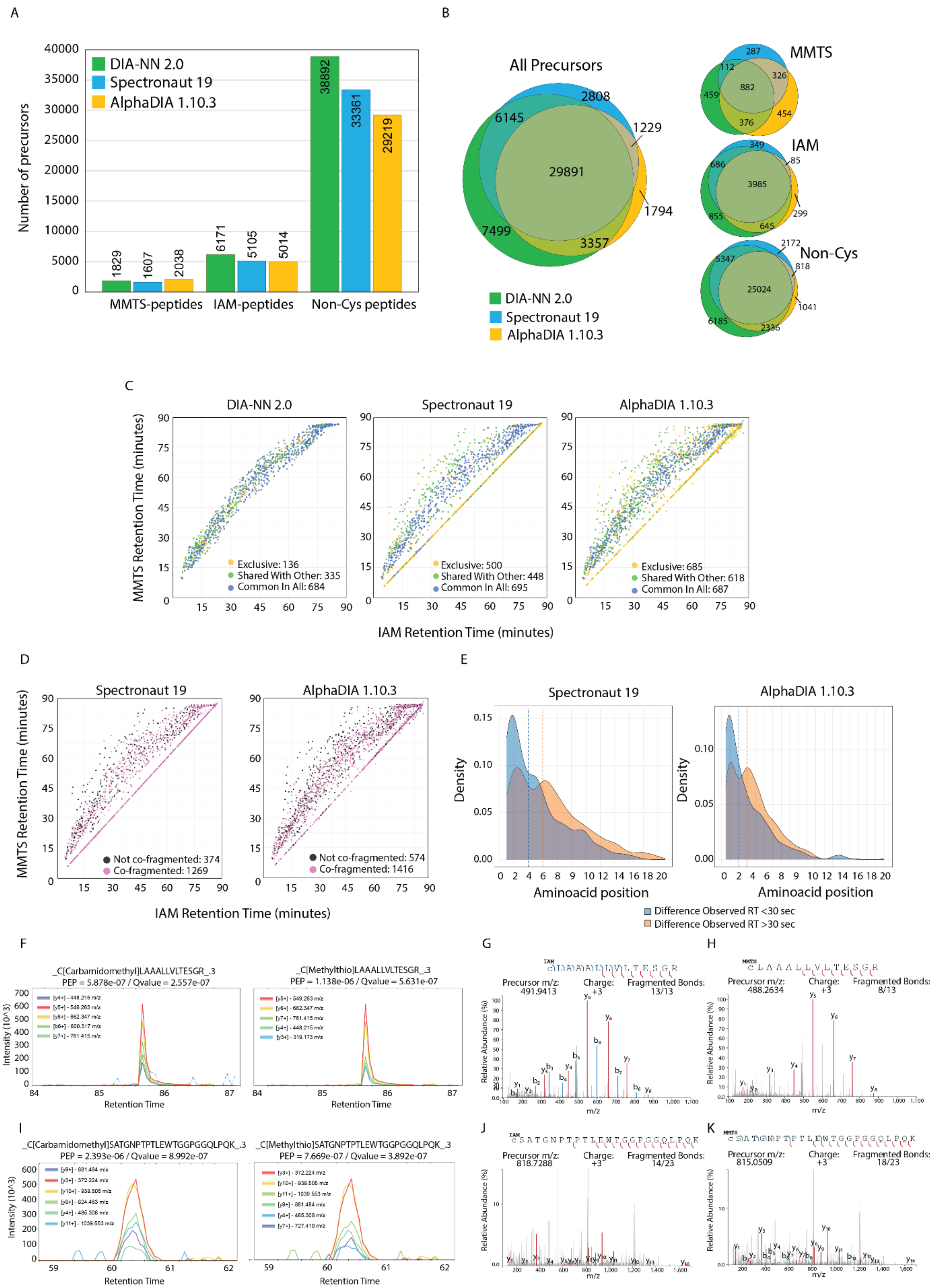

Supplementary Figure S2. *(A) Number of identified precursors in two raw files from porcine atrial tissue processed using the PACREDOX protocol in three different DIA search engines without the use of experimental libraries: green - DIA-NN 2.0, blue - Spectronaut 19, yellow - AlphaDIA 1.10.3. (B) Euler diagrams showing the overlap between identified precursors in DIA-NN, AlphaDIA and Spectronaut*

searches, either non-Cys containing peptides, IAM-labelled or MMTS-labelled peptides. (C) Scatter plot of retention times (RT) from IAM-labelled peptides vs their MMTS-labelled counterparts in an 88 minutes LC-run in DIANN, Spectronaut and AlphaDIA. Colors of the dots indicate whether that peptide pair was identified only in that search engine (yellow), in that search engine and another (green) or identified by the three search engines (blue). (D) Scatter plot of retention times (RT) from IAM-labelled peptides vs their MMTS-labelled counterparts in an 88 minutes LC-run in Spectronaut and AlphaDIA. Colors of the dots indicate whether the peptide pair can be potentially co-isolated and co-fragmented in the same DIA window (pink). (E) Density plots showing the position of the labelled cysteine in the peptide (starting from the N-termini) for peptide identified in the same retention time window (blue), or not (orange). Dashed lines represent the median value of each distribution. (F) Extracted Ion Chromatogram of an IAM-labeled peptide and a wrongly assigned MMTS-labeled peptide as reported in Spectronaut. (G-H) Annotated fragmentation spectrum at the apex as reported in Spectronaut of the peptides in F. (I) Extracted Ion Chromatogram of an MMTS-labeled peptide and a wrongly assigned IAM-labeled peptide as reported in Spectronaut. (J-K) Annotated fragmentation spectrum at the apex as reported in Spectronaut of the peptides in I. Spectrum plots from panels G, H, J and K were generated using the Interactive Peptide Spectral Annotator tool [1].

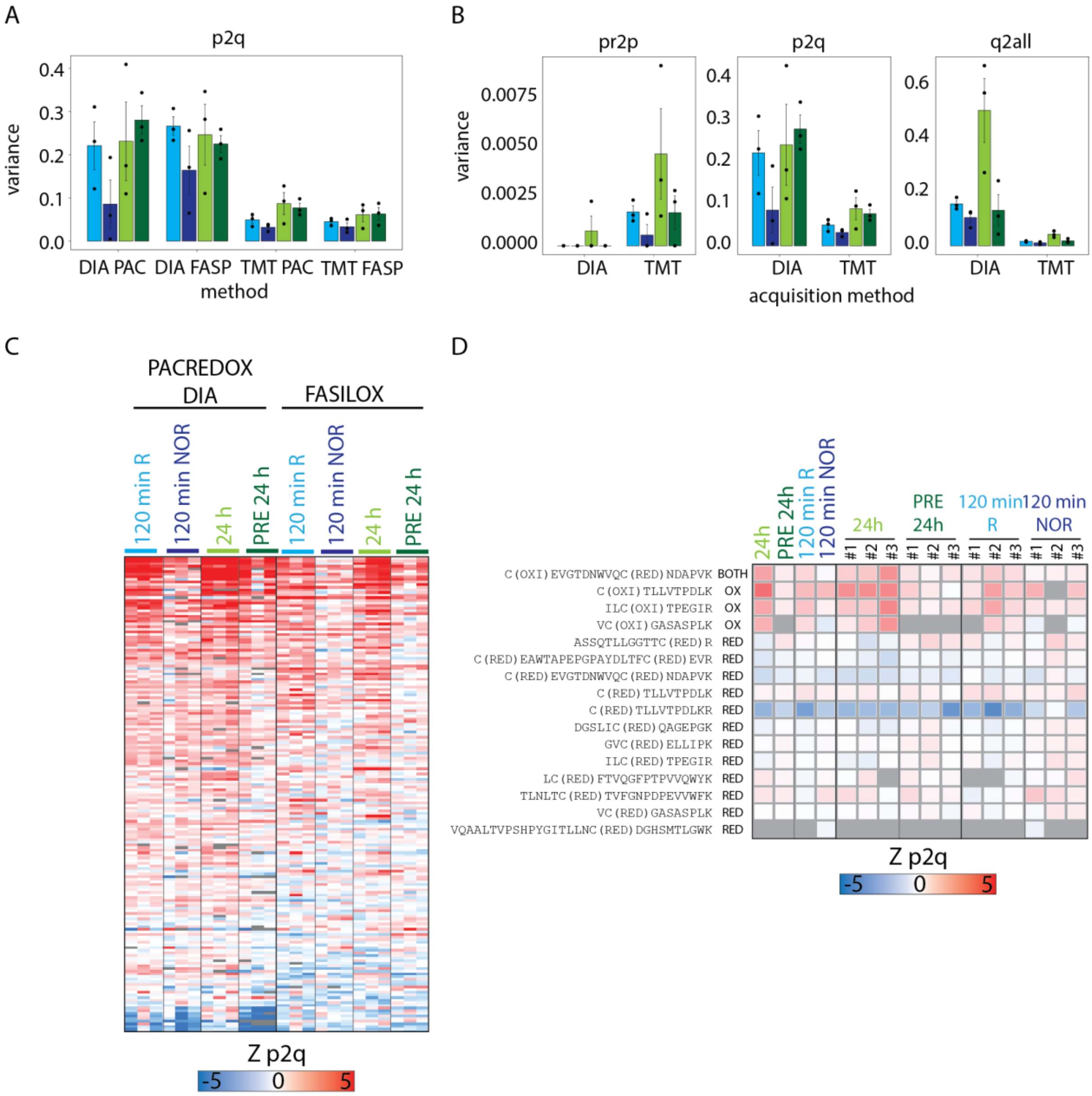

Supplementary Figure S3. *(A) Variances from four different experimental strategies (PAC-DIA, PAC-TMT, FASP-DIA, FASP-TMT) calculated in the peptide to protein integration level in iSanXot. The bars represent the mean ± SEM of n=3 biological replicates. Each colour indicates a different biological comparison (all of them are relative to baseline condition). (B) Variances from PAC-DIA or PAC-TMT quantification strategies calculated in the three integration levels (precursor or scan to peptide – pr2p, peptide to protein – p2q and protein to all – q2all) in iSanXot. Height of the bar is the average of n=3 biological replicates, and the error bar represents the standard deviation of the mean. Each colour indicates a different biological comparison (all of them are relative to baseline condition). (C) Heatmap of Zp2q values for MMTS-labelled peptides common in PACREDOX with DIA and FASILOX analysis. (D) Heatmap of Zp2q values for reduced and oxidized Cys-containing peptides from MYOM2 protein detected exclusively by DIA analysis.*

A

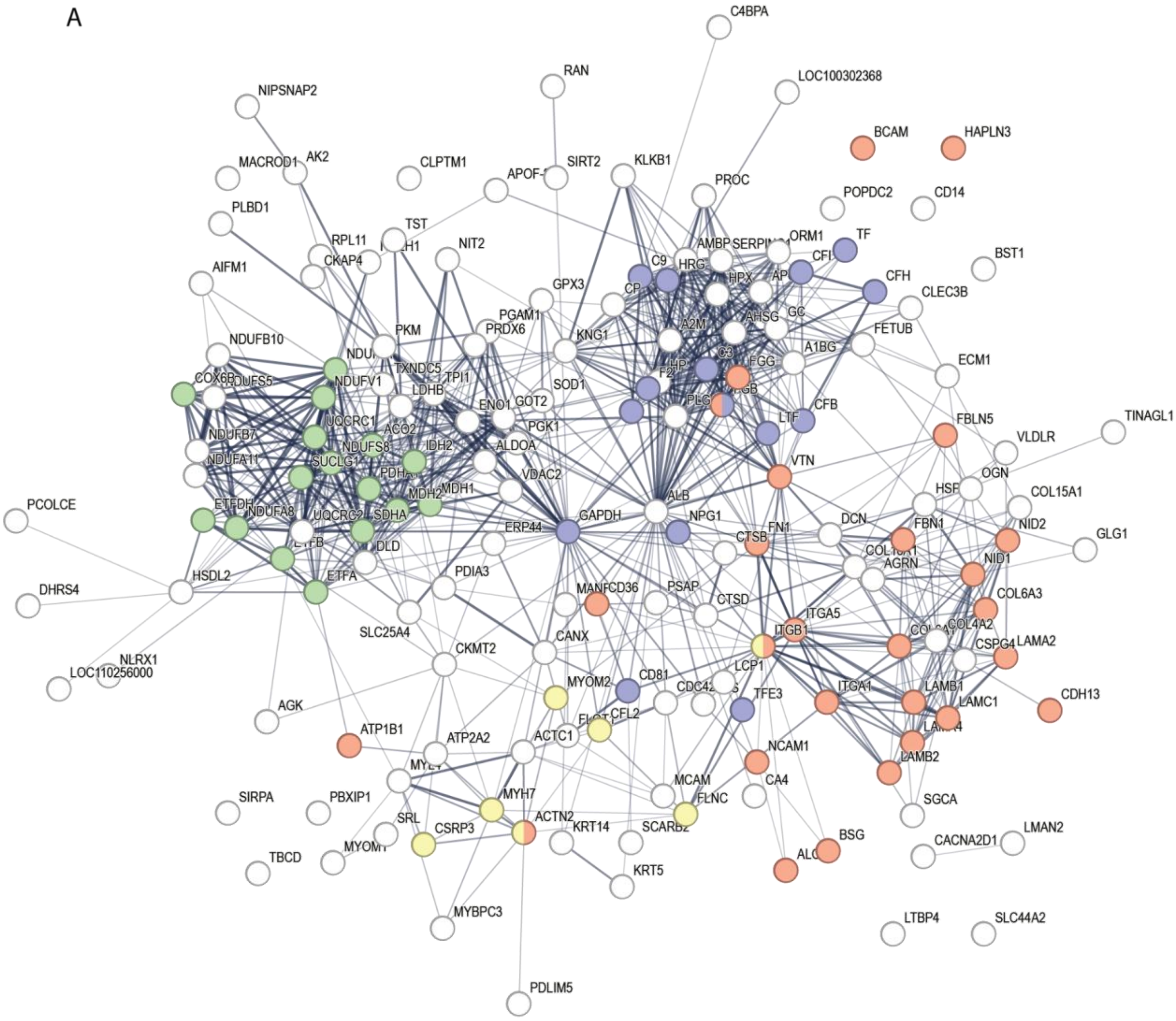

B

Gene Ontology Term	FDR
Cellular Respiration	1.6 e-2
Humoral Immune Response	1.3 e-4
Cell Adhesion	1.8 e-4
Sarcomere Organization	4.6 e-2

Supplementary Figure S4. *(A) STRING [2] network of proteins containing reversibly oxidized Cys detected in the PAREDOX DIA analysis. Colour represents whether the protein belongs to the Gene Ontology (GO) terms from panel B. (B) Most representative GO terms from the network shown in A, together with the FDR-corrected significance value for over-representation corrected by the measured proteomic background.*

A

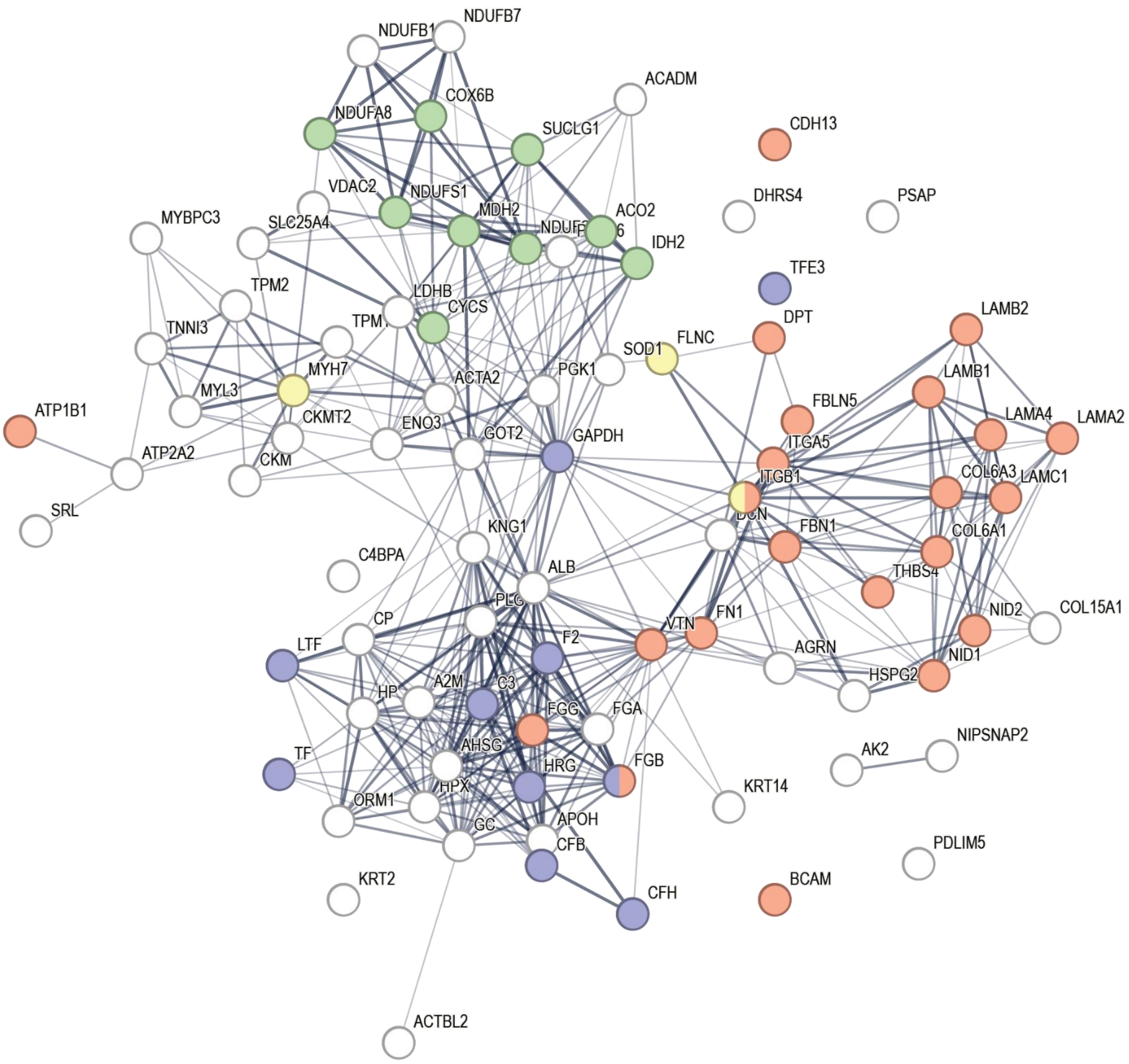

B

Gene Ontology Term	FDR
Cellular Respiration	ns.
Humoral Immune Response	1.6e-3
Cell Adhesion	1.9e-6
Sarcomere Organization	n.s.

Supplementary Figure S5. *(A) STRING [2] network of proteins containing reversibly oxidized Cys detected in the FASILOX analysis. Colour represents whether the protein belongs to the Gene Ontology (GO) terms from panel B. (B) Most representative GO terms from the network shown in A, together with the FDR-corrected significance value for over-representation corrected by the measured proteomic background. "n.s." stands for not-significant.*

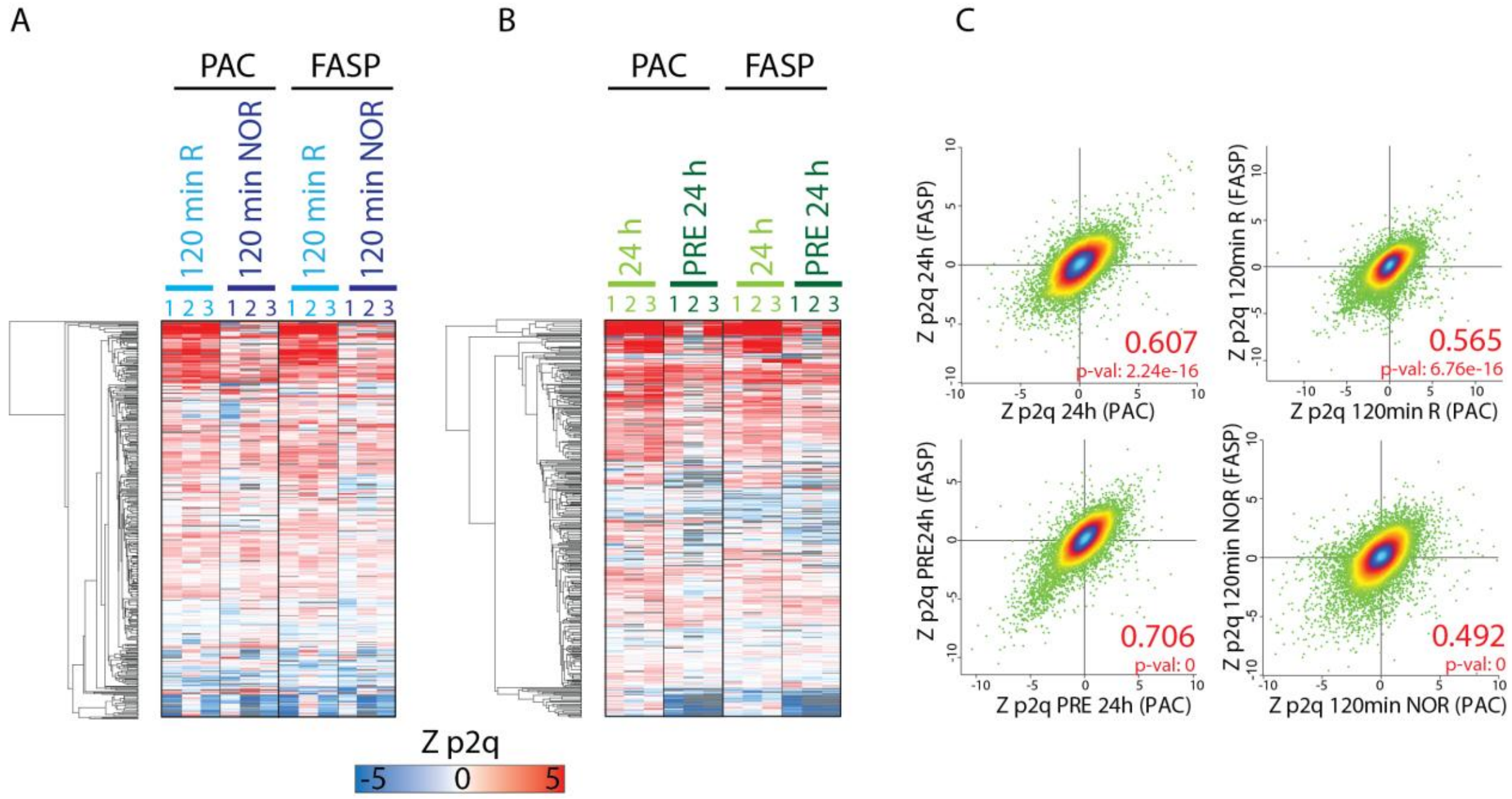

Supplementary Figure S6. *(A) Heatmap of Zp2q values for MMTS-labelled peptides common in FASP-redox and PAC-redox samples analysed using DIA for 120 min R and 120 min NOR samples (relative to baseline). (B) Heatmap of Zp2q values for MMTS-labelled peptides common in FASP-redox and PAC-redox samples analysed using DIA for 24 h and PRE 24 h samples (relative to baseline). (C) Correlation plots of Zp2q values calculated for all peptides in PAC versus FASP-based digestion experiments, both analysed using DIA. Colour indicates the density of the point distribution. Red value on the bottom right corner indicates the Person correlation of the distribution, and below the correlation p-value.*

References

(1) Brademan, D. R.; Riley, N. M.; Kwiecien, N. W.; Coon, J. J. Interactive Peptide Spectral Annotator: A Versatile Web-Based Tool for Proteomic Applications. *Mol Cell Proteomics* **2019**, *18* (8 suppl 1), S193-S201. DOI: 10.1074/mcp.TIR118.001209 From NLM Medline.

(2) Szklarczyk, D.; Kirsch, R.; Koutrouli, M.; Nastou, K.; Mehryary, F.; Hachilif, R.; Gable, A. L.; Fang, T.; Doncheva, N. T.; Pyysalo, S.; Bork, P.; Jensen, L. J.; von Mering, C. The String Database in 2023: Protein-Protein Association Networks and Functional Enrichment Analyses for Any Sequenced Genome of Interest. *Nucleic Acids Res* **2023**, *51* (D1), D638-D646. DOI: 10.1093/nar/gkac1000 From NLM Medline.
